# Identifying Discriminative Biological Function Features and Rules for Cancer-Related Long Non-coding RNAs

**DOI:** 10.3389/fgene.2020.598773

**Published:** 2020-12-16

**Authors:** Liucun Zhu, Xin Yang, Rui Zhu, Lei Yu

**Affiliations:** ^1^School of Life Sciences, Shanghai University, Shanghai, China; ^2^Department of Medical Oncology, Shanghai Concord Medical Cancer Center, Shanghai, China

**Keywords:** decision rule, KEGG pathway, gene ontology, decision tree, long non-coding RNAs, cancer

## Abstract

Cancer has been a major public health problem worldwide for many centuries. Cancer is a complex disease associated with accumulative genetic mutations, epigenetic aberrations, chromosomal instability, and expression alteration. Increasing lines of evidence suggest that many non-coding transcripts, which are termed as non-coding RNAs, have important regulatory roles in cancer. In particular, long non-coding RNAs (lncRNAs) play crucial roles in tumorigenesis. Cancer-related lncRNAs serve as oncogenic factors or tumor suppressors. Although many lncRNAs are identified as potential regulators in tumorigenesis by using traditional experimental methods, they are time consuming and expensive considering the tremendous amount of lncRNAs needed. Thus, effective and fast approaches to recognize tumor-related lncRNAs should be developed. The proposed approach should help us understand not only the mechanisms of lncRNAs that participate in tumorigenesis but also their satisfactory performance in distinguishing cancer-related lncRNAs. In this study, we utilized a decision tree (DT), a type of rule learning algorithm, to investigate cancer-related lncRNAs with functional annotation contents [gene ontology (GO) terms and KEGG pathways] of their co-expressed genes. Cancer-related and other lncRNAs encoded by the key enrichment features of GO and KEGG filtered by feature selection methods were used to build an informative DT, which further induced several decision rules. The rules provided not only a new tool for identifying cancer-related lncRNAs but also connected the lncRNAs and cancers with the combinations of GO terms. Results provided new directions for understanding cancer-related lncRNAs.

## Introduction

Cancer has been a major public health problem worldwide for many centuries (Siegel et al., 2016). Cancer is defined as a group of diseases that are characterized by disordered cell proliferation and invasion into normal tissues. Although the cure of cancer has not been discovered yet, research on understanding this complex disease has progressed considerably. Genetic alterations were thought to be the main cause of cancer initiation and progression in classical theory (Vogelstein et al., 1988). At present, cancer is viewed as a complex disease associated with accumulative genetic mutation, epigenetic aberration, chromosomal instability, and expression alteration. The discovery of genetic code for protein-coding genes can accelerate research on oncogenic genes or tumor suppressors that participate in tumorigenesis. This phenomenon revolutionized the understanding of how genetic alterations contribute to the abnormal phenotypes of cancer. However, previous studies aimed to identify oncogenes by focusing on protein-coding sequences, which account for a very small part of all transcripts (Birney et al., 2007). Increasing lines of evidence suggest that many non-coding transcripts, which are termed as non-coding RNAs (ncRNAs), have important regulatory roles in cancer (Calin et al., 2007; Carninci and Hayashizaki, 2007; Pan and Shen, 2019; Pan et al., 2019).

Messenger RNAs (mRNAs) are a small fraction of the RNA population and an intermediate between DNA and protein in the translation of genetic information into diverse biological processes. Many ncRNAs cannot be translated into proteins but can still directly function as regulatory elements (Khalil et al., 2009). According to the length of transcripts, these ncRNAs can be divided into two subgroups, namely, small ncRNAs with length less than 200 bp, including microRNAs and siRNAs, and long non-coding RNAs (lncRNAs) with length higher than 200 bp. With the rapid development of detection technologies, such as whole-transcripts sequencing, more than 50,000 lncRNAs have been identified, which account for the majority of human transcriptome and have attracted increasing research attention in recent years (Iyer et al., 2015; Mirza et al., 2015; Pan and Xiong, 2015).

In the early 1980s, scientists discovered lncRNAs by screening cDNA libraries and identified several milestone lncRNAs, such as XIST and H19; however, the term lncRNA has not been proposed at that time (Bartolomei et al., 1991; Brown et al., 1992). Although this new class of RNA lacks the ability to encode proteins, lncRNAs exhibit diversity and complexity in biological structures and functions. The biological roles of lncRNAs are mainly attributed to the following aspects: *cis* or *trans* regulation of transcription, modulation of mRNA or protein activity, and nuclear organization (Geisler and Coller, 2013; Cao et al., 2018). For example, lncRNAs (e.g., GAS5) serve as a decoy and can bind to target gene promoters to suppress functional activation (Wang and Chang, 2011). Some lncRNAs, including AIR and CCND1, perform distinct and effective interactions with protein complexes and guide them to the specific target locus for gene regulation (Ma et al., 2013). In particular, lncRNAs play crucial roles in tumorigenesis and serve as oncogenic factors or tumor suppressors (Tsai et al., 2011). The aberrant and specific expression of lncRNAs in various tumors has revealed their potential new participation in cancer development. For instance, lncRNA aHIF, which is transcribed from the genomic location 14q23.2, is over-expressed in renal and breast cancers and shows high correlation with poor prognosis (Thrash-Bingham and Tartof, 1999; Cayre et al., 2003). Another lncRNA called MEG3 is involved in cervical and bladder cancers by promoting cell proliferation *via* the induction of p53-mediated transactivation (Zhu et al., 2015). Moreover, lncRNA MALAT1 is conserved among vertebrates and plays an important role in cell proliferation; the depletion of this RNA can cause an inhibitory effect on breast cancer, thereby contributing to tumor progression (Jadaliha et al., 2016).

Given the critical roles of lncRNAs in cancer, they could be used as novel diagnostic biomarkers and therapeutic targets for cancer treatments (Crea et al., 2014). A large number of lncRNAs have been identified as potential regulators in tumorigenesis by using traditional experimental methods; however, such methods are time consuming and expensive due to tremendous amount of lncRNAs needed. It is an alternative way of designing effective computational methods (Zhao et al., 2015; Chen et al., 2017c; Yuan et al., 2018). However, these methods demonstrate poor interpretability. Although these methods can provide satisfactory performance, their principles are difficult to capture, leading to limited biological and medical insights. In the present study, we adopted a rule learning algorithm, namely, decision tree (DT) (Safavian and Landgrebe, 1991), to analyze cancer-related lncRNAs, which were obtained from a previous study (Zhao et al., 2015). These and other lncRNAs were encoded using functional annotation contents [gene ontology (GO) terms and KEGG pathways] of their co-expressed genes. The DT algorithm was applied on such dataset, in which lncRNAs were represented by essential features and filtered by some feature selection methods, to construct a large DT and extract several decision rules. These rules clearly indicated the combination of GO terms that could identify cancer-related lncRNAs and presented a clear overview of the functional annotation contents on cancer-related lncRNAs. The rules could also be used as a classifier for identification of cancer-related lncRNAs but have lower performance than other black-box classifiers.

## Materials and Methods

### Datasets

In a previous study (Zhao et al., 2015), 70 cancer-related lncRNAs were manually validated and collected from the lncRNA Disease database (Chen et al., 2013; Bao et al., 2019) and published literature. Of these lncRNAs, 57 were expressed in the Illumina Body Map (Farrell et al., 2014) and were selected as positive samples. Meanwhile, 14,829 lncRNAs were retrieved from the LNCipedia database (Volders et al., 2013, 2019); none of which were reported or confirmed to be associated with tumorigenesis. These lncRNAs were temporarily treated as negative samples due to the lack of evidence that they are cancer related. The detailed information of the selected positive and negative samples can be found in our previous study (Chen et al., 2017c). The number of the negative samples was higher than that of the positive samples, i.e., the dataset is imbalanced, with sample ratio of approximately 1:260.

### Feature Extraction With GO and KEGG Pathway

Similar to previous studies (Chen et al., 2017c; Yuan et al., 2018), we employed enrichment theory (Carmona-Saez et al., 2007) to encode lncRNAs. Each lncRNA in the dataset was represented by a feature vector with 19,090 elements, in which 18,803 and 287 represent the enrichment scores of GO and KEGG pathway, respectively. The computing processes of the two kinds of enrichment scores were described below.

Given one lncRNA *x* in the dataset, let *G*(*x*) be a set of co-expressed genes with *x*, where the identity of the co-expressed genes can be found in previous studies (Chen et al., 2017c; Yuan et al., 2018). The GO enrichment score of one GO term *g*_j_ and lncRNA *x* can be calculated as follows:

(1) $$S_{\text{GO}}\left(x,g_{\text{j}}\right)=-log_{10}\left(\sum_{l=m}^{\text{n}}\frac{\left(\begin{array}{c} M\\ l \end{array}\right)\left(\begin{array}{c} N-M\\ n-l \end{array}\right)}{\left(\begin{array}{c} N\\ n \end{array}\right)}\right)$$

where *N* represents the total number of human genes, *M* denotes the number of genes annotated to *g*_j_ in the GO database, *n* refers to the number of genes in *G*(*x*), and *m* indicates the number of genes in *G*(*x*) that are also annotated to *g*_j_. Thus, the high GO enrichment score *S*_GO_ indicates a strong association between an lncRNA and a GO term.

Given one lncRNA *x* and a KEGG pathway *k*_j_, the KEGG enrichment score is calculated as follows:

(2) $$S_{\text{KEGG}}\left(x,k_{\text{j}}\right)=-log_{10}\left(\sum_{l=m}^{\text{n}}\frac{\left(\begin{array}{c} M\\ l \end{array}\right)\left(\begin{array}{c} N-M\\ n-l \end{array}\right)}{\left(\begin{array}{c} N\\ n \end{array}\right)}\right)$$

where *N* and *n* have the same definitions as those in Eq. 1, *M* represents the number of genes in *k*_j_based on the KEGG database, and *m* denotes the number of genes in both *G*(*x*) and *k*_j_. Here, the high KEGG enrichment score *S*_KEGG_ indicates a strong relationship between a lncRNA and a KEGG pathway.

### Feature Selection of Minimum Redundancy Maximum Relevance (mRMR)

Many enrichment scores were used to represent each lncRNA and indicate the relationship between a lncRNA and GO term or KEGG pathway. Obviously, it is impossible that all GO terms and KEGG pathways give same contribution of describing cancer-related lncRNAs. An effective feature selection procedure is necessary.

Here, we employed a powerful and widely used feature selection method, namely, mRMR (Ding and Peng, 2005; Peng et al., 2005; Chen et al., 2017b, 2018; Radovic et al., 2017; Zhao et al., 2018). This approach consists of two parts: minimum redundancy among features and maximum relevance between features and class labels. Thus, the essential features extracted by mRMR method can construct a compact feature subspace, that is, less features can hold more essential information and provide higher classification performance. These parameters are all measured using mutual information (MI). For two variables *x* and *y*, their MI is calculated by,

(3) $$I\left(x,y\right)=\iint p\left(x,y\right)\log\frac{p\left(x,y\right)}{p\left(x\right)p\left(y\right)}dxdy$$

where *p*(*x*) stands for the marginal probabilistic density of *x* and *p*(*x*,*y*) represents the joint probabilistic density of *x* and *y*. Generally, a high MI value indicates the high relevance of two variables. The importance of one feature is evaluated by its relevance to the class label and its redundancy to other features. To exhibit the importance of all features, this method outputs an mRMR feature list, in which all features are sorted according to their importance. Features with minimum redundancy and maximum relevance will have high ranks. To obtain such list, a loop procedure is executed. Initially, this list is empty. In each round, a feature with maximum relevance to the class label and minimum redundancy to features in the current list is selected and appended to the list. When all features have been in the list, the loop stops.

We adopted the mRMR program developed by Peng et al. (2005) which can be retrieved from http://penglab.janelia.org/proj/mRMR/. Default parameters were used for convenience.

### Incremental Feature Selection

We obtained a feature list by using mRMR method. The optimal feature subspace for a given classification algorithm is still difficult to determine. To this end, incremental feature selection (IFS) (Liu and Setiono, 1998), another method based on a supervised classifier, was adopted. A series of feature subsets is first constructed from an existing feature list (e.g., mRMR feature list). The first feature subset contains the top feature, the second feature subset contains the top two features, and so on. For each feature subset, a classifier (i.e., DT in this study) is constructed on the samples represented by features from the feature subset whose performance is further evaluated using 10-fold cross-validation (Kohavi, 1995). After assessing all the feature subsets, the feature subset with the highest performance measured by Matthew correlation coefficients (MCCs) (Matthews, 1975) is selected as the optimum feature subset. The classifier with this feature subset is termed as the optimum classifier.

### DT

Decision Tree (Safavian and Landgrebe, 1991) is a popular and classic machine learning algorithm and a non-parametric supervised learning method for classification and regression. This algorithm is important because it can produce rules that are simple to understand and interpret, thereby yielding more clues for the investigated problems than other black-box classifiers. In addition, the performance of DT is satisfactory in many cases.

**FIGURE 1:**
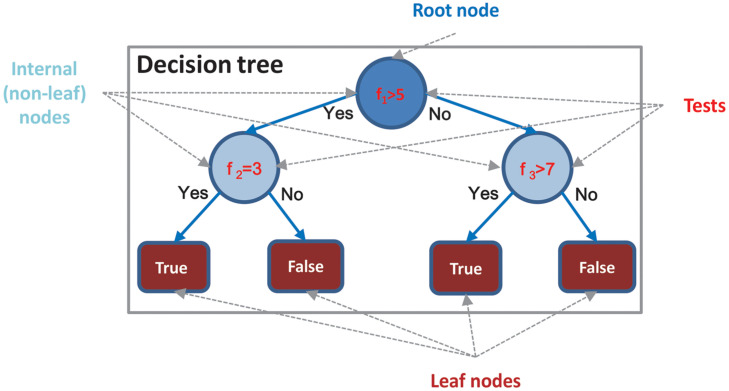
Example of DT.

Decision Tree is represented by a flowchart-like structure. One simple example is illustrated in Figure 1. Each internal (i.e., non-leaf) node of the tree corresponds to some input variables/features, which are basically a decision maker. Each terminal (i.e., leaf) node of the tree represents a class label, which is the decision outcome. A common strategy for constructing a DT is top–down induction [6], which is a greedy algorithm. The key procedure in creating a DT is determining an optimal way for splitting internal nodes. Different schemes with different metrics, such as Gini index, information gain, and information gain ratio, have been proposed to build different types of DTs.

In this study, we built a DT with Scikit-learn (Pedregosa et al., 2011), a machine learning tool in Python. Scikit-learn uses an optimized version of the CART algorithm and constructs DTs with a scheme utilizing the Gini index. From the DT, decision rules can be generated by using a path from the root and terminal nodes, which can be represented as follows:

*if* conditions 1, 2, *and* 3, *then* outcome,

where each condition is the test result of a feature in the internal node, and the outcome is the class label indicated by the corresponding leaf node. With the obtained rules, the combination of features that are important for describing cancer-related lncRNAs can be easily accessed. Such features can be an essential biomarker for determining cancer-related lncRNAs.

### SMOTE

As indicated in section “Datasets,” the analyzed dataset consists of different numbers of positive and negative samples (i.e., cancer-related lncRNAs and lncRNAs not related to cancer). Building an efficient classifier on such an imbalanced dataset is difficult. Thus, we adopted the synthetic minority over-sampling technique (SMOTE) (Chawla et al., 2002) to produce balanced data before constructing the classifier. SMOTE is an oversampling method that has wide applications (Li et al., 2014; Marques et al., 2016; Wang et al., 2018; Zhang et al., 2019). SMOTE aims to produce new samples for the minor sample class (i.e., positive samples in this study) iteratively until the size of the minor sample class is equal to that of the major sample class (i.e., negative samples in this study).

In this work, we used the tool “SMOTE” in Weka (Witten and Frank, 2005) to produce new positive samples. The main parameter, which determines the number of nearest neighbors, was set to five. Finally, the numbers of positive and negative samples were equivalent. Because the newly produced samples may influence the feature selection results, these samples were produced after the mRMR method was used to evaluate the importance of features. And the SMOTE was only adopted in the procedure of evaluating the performance of DT.

### Performance Evaluation

We mainly evaluated the prediction performance of the constructed classifiers by using MCC (Matthews, 1975; Chen et al., 2017a; Cui and Chen, 2019; Zhao R. et al., 2019; Zhao X. et al., 2019) through 10-fold cross-validation (Kohavi, 1995; Che et al., 2020; Jia et al., 2020; Liang et al., 2020; Zhou et al., 2020) because the investigated dataset was imbalanced and MCC is a balanced measurement even if the class sizes differ. The MCC can be calculated by the following equation:

(4) $$MCC=\frac{TP\times TN-FP\times FN}{\sqrt{\left(TN+FN\right)\times \left(TN+FP\right)\times \left(TP+FN\right)\times \left(TP+FP\right)}}$$

where TP stands for true positive, FP represents false positive, FN denotes false negative and TN indicates true negative. The range of MCC is between -1 and +1. The classifier will be good when MCC approaches +1.

In addition, we employed five other measurements for reference, namely, sensitivity (SN), specificity (SP), prediction accuracy (ACC), Recall, Precision, and F1-measure. They can be computed by the following set of equations:

(5) $$\left\{ \begin{array}{c} SN=\frac{TP}{TP+FN}\\ SP=\frac{TN}{TN+FP}\\ ACC=\frac{TP+TN}{TP+FN+FP+TN}\\ Precision=\frac{TP}{TP+FP}\\ F1-measure=\frac{2\times Precision\times Recall}{Precision+Recall} \end{array}\right.$$

where Recall is same as SN.

## Results

**FIGURE 2:**
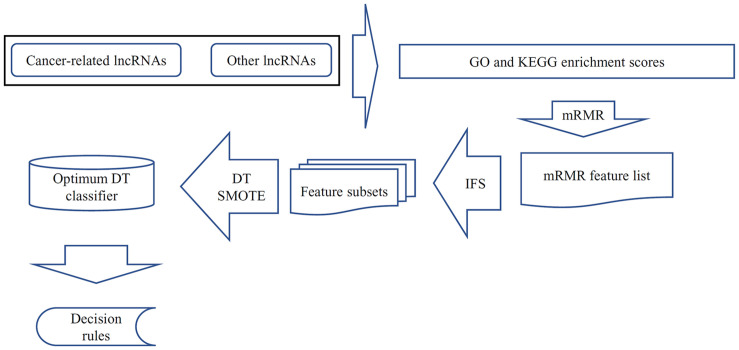
Analysis of cancer-related lncRNAs. All lncRNAs were encoded using the GO and KEGG enrichment scores, which are analyzed *via* the mRMR feature selection method and result in the mRMR feature list. The IFS method is applied on this list with the help of DT and SMOTE. Finally, the DT classifier with the best performance is obtained and further used for constructing the decision rules.

In this study, we used several machine learning algorithms to investigate cancer-related lncRNAs. This work aimed to build a classifier for identifying cancer-related lncRNAs and provide a clear outline of the functional contents of cancer-related lncRNAs. The procedures are illustrated in Figure 2. This section presents the results in each step.

### Results of mRMR

Each investigated lncRNA was represented by many GO and KEGG-based features. We first used mRMR to evaluate these features. The output mRMR feature list was selected for subsequent analysis and is provided in Supplementary Material 1.

### Results of IFS Incorporating DT

**FIGURE 3:**
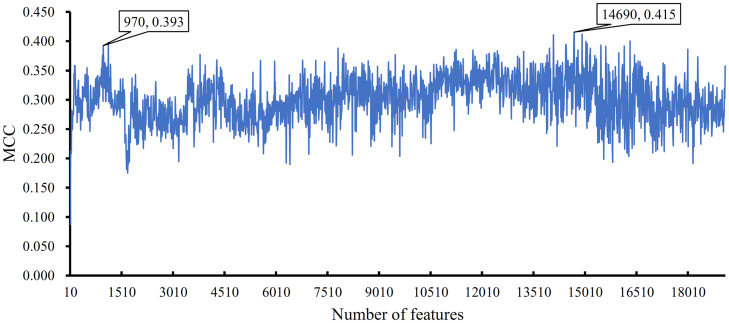
IFS curve to show the change in MCC with different numbers of top features in the mRMR feature list. The highest MCC of 0.415 is obtained when the top 14,690 features are used. However, the MCC is still high (0.393) when the top 970 features are adopted.

The importance of features is indicated by their ranks in the mRMR feature list. The combination of some top features can be the optimum feature subspace in a given classification algorithm. To this end, IFS method was employed. However, this method was time consuming if all possible feature subsets were considered. Thus, we used step 10 to construct feature subsets. In this method, the top ten features in the list constituted the first feature subset, the top 20 features comprised the second subset, and so on. All lncRNAs were encoded by features in each constructed feature subset, on which a DT classifier was built. Tenfold cross-validation was adopted to evaluate the performance of such classifier. The predicted results are provided in Supplementary Material 2. An IFS curve was plotted (Figure 3) to easily observe the change in MCC with different numbers of top features. When the top 14,690 features were used, the DT classifier yielded the maximum MCC value of 0.415. Thus, we termed the DT classifier with these 14,690 features as the optimum classifier. Other three measurements, namely, SN, SP, ACC, Precision, and F1-measure are listed in Table 1 and had values of 0.702, 0.992, 0.991, 0.161, and 0.240, respectively. The SP was higher than the SN because cancer-related lncRNAs (positive samples) were significantly less than other lncRNAs (negative samples).

**TABLE 1:** Performance of some key DT classifiers.

Classifier	SN	SP	ACC	MCC	Precision	F1-measure
Optimum DT classifier	0.702	0.992	0.991	0.415	0.161	0.240
DT classifier with the top 970 features	0.737	0.990	0.989	0.393	0.216	0.320

The optimum DT classifier adopted too many features, thereby decreasing its efficiency. By carefully checking the IFS curve in Figure 3 and MCCs in Supplementary Material 2, we found that the DT classifier still obtained satisfactory performance with an MCC of 0.393 (e.g., guarantee the trade-off between the number of features and performance) when the top 970 features were used. The SN, SP, ACC, Precision, and F1-measure were 0.737, 0.990, 0.989, 0.216, and 0.320, respectively. The detailed performance is listed in Table 1. The performance of the SN even exceeded that of the optimum DT classifier. The performance of these classifiers was almost at the same level. Accordingly, the DT classifier was appropriate for real applications.

We selected the DT classifier with the top 970 features as the proposed classifier. The DT was executed on all lncRNAs again to build decision rules, which are listed in Supplementary Material 3. A total of 219 rules were obtained. By analyzing these rules, we obtained a clear picture of the combination of features that are essential for determining cancer-related lncRNAs. We also revealed differences between cancer-related lncRNAs and other general lncRNAs. An extensive discussion will be given in section “DISCUSSION.”

### Comparison of Previous Classifiers

**FIGURE 4:**
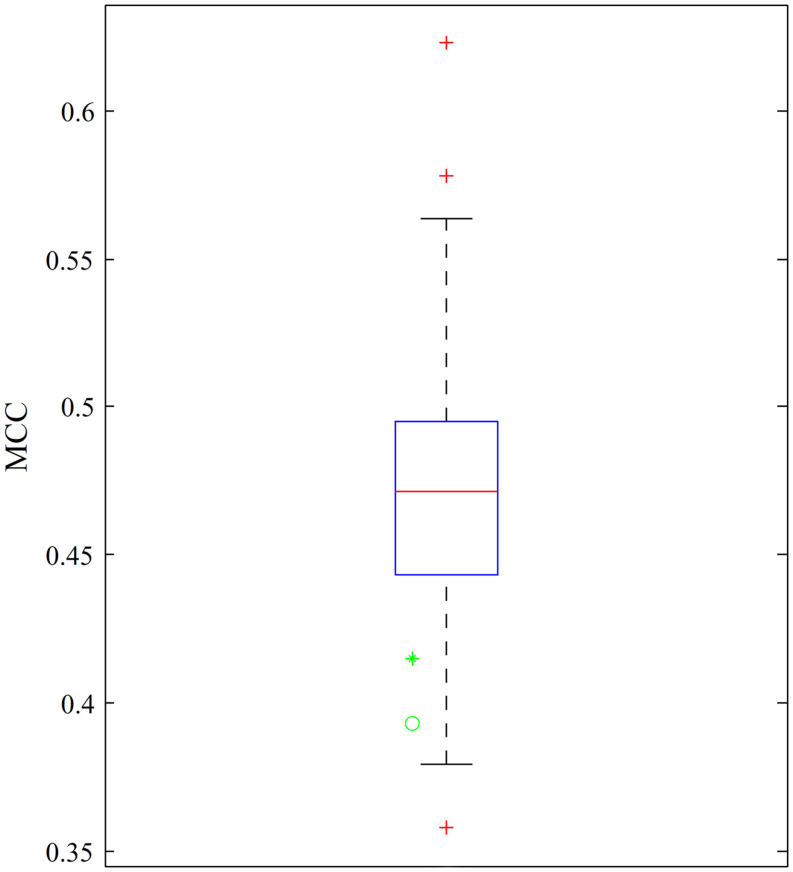
Boxplot demonstrating the MCCs yielded by the previous classifiers and our classifiers. MCCs yielded by our classifiers are marked with a green star and a green cycle to indicate the MCC of the optimum DT classifier and the MCC of the DT classifier with the top 970 features, respectively.

Chen et al. (2017c) adopted a complicated scheme to tackle the imbalanced dataset by dividing the negative samples into 130 subsets. Each negative sample subset combined with the positive sample set constitutes a balanced dataset. A dagging classifier (Ting and Witten, 1997) with support vector machine and optimum GO and KEGG enrichment features was built in each dataset. MCCs yielded by 10-fold cross-validation are shown in Figure 4, which also provides the MCCs of the optimum DT and DT classifiers with the top 970 features. The obtained MCCs were at the bottom of the box, indicating the lower performance of the two classifiers than that of previous classifiers but still better than some of the previous classifiers. Furthermore, previous classifiers were absolute black-box classifiers and provided limited clues for determining differences between cancer-related lncRNAs and other lncRNAs. However, our classifiers could output decision rules, as listed in Supplementary Material 3, and provide additional insights.

## Discussion

As a novel type of ncRNAs, lncRNAs play important regulatory roles in gene expression (Kapranov et al., 2007). Emerging evidence has confirmed the close relationship between lncRNAs and cancers (Guttman et al., 2009; Huarte et al., 2010). This finding has inspired investigators to explore the biological mechanism of tumorigenesis driven by certain lncRNAs. The first key task in investigating the tumor-related functions of lncRNAs is to identify lncRNA signatures that contribute to the initiation or progression of tumorigenesis. Considering that tumor-associated genes can be categorized as oncogenes and tumor suppressor genes (Croce, 2008), we aimed to build not only a classifier for distinguishing lncRNAs related or unrelated to tumors but also reveal additional information about the essential characteristics of cancer-related lncRNAs.

Several machine learning algorithms, including mRMR, IFS, DT, and SMOTE, were applied in the construction of a DT classifier, which can identify cancer-related lncRNAs with an MCC value of 0.393 based on 970 function features. Few manually validated lncRNAs were implicated in tumorigenesis but were still insufficient to consist of a set of positive samples for model training. Hence, the number bias between the positive and negative samples would result in a slightly inferior performance of the prediction model. However, our study provided an effective and novel analysis pipeline to capture the essence of tumor-related lncRNAs through their correlated mRNAs and functional annotations. In addition, the decision rules yielded supplied an extended explanation on how certain lncRNAs affect tumorigenesis. These interpretable rules could highlight crucial functions as a set of GO terms or KEGG pathways, which may have been neglected in previous studies but require deep investigation for candidate tumorigenesis roles. Recent publications in several experimental journals present some consistent rules. Among the 219 decision rules, 42 rules were used to identify cancer-related lncRNAs and the 177 remaining rules could exclude cancer-related lncRNAs. Thus, 219 rules were divided into two groups. We selected some decision rules from each group as examples to give a detailed discussion below.

### Rules for Cancer-Related lncRNA Identification

The first rule of cancer recognition was Rules_66 involving 48 GO terms. Apart from the general GO terms, such as GO: 0009301 (snRNA transcription) and GO: 0051861 (glycolipid binding), we also identified a group of effective GO terms that contribute to the identification of cancer-related lncRNA.

GO: 0043849 describes the Ras palmitoyltransferase activity, was used to construct this rule. According to recent publications, Ras palmitoyltransferase can participate in lipid metabolism and epithelial–mesenchymal transition in breast cancer cells *via* lncRNA-associated regulatory pathways (Barnard, 2014). Therefore, the prediction of such GO term as a candidate enrichment cluster for cancer-related lncRNAs is reasonable. Apart from GO: 0043849, the next GO term GO: 0006275, is a general parameter for various rules and describes the regulation of DNA replication. GO: 0006275, describing a biological process is negatively enriched with cancer-related lncRNAs. Given that lncRNAs contribute to the regulation of cell proliferation and their differentiation are generally downregulated (Zhou et al., 2015; Bian et al., 2016), identifying this parameter as a potential cancer-associated GO term with low enrichment level is reasonable. Another GO term GO: 0090162, which describes the establishment of epithelial cell polarity, was also used as a general parameter for classification. This GO term contributes to the identification of cancer-related lncRNAs. Cell polarity, especially the epithelial cell polarity, is an important feature for distinguishing normal cells from tumor and stem cells. The loss of cell polarity is generally regarded as a significant biomarker for tumorigenesis and us regulated by various cancer-related lncRNAs (McCaffrey and Macara, 2011; Royer and Lu, 2011; Martin-Belmonte and Perez-Moreno, 2012). Therefore, the enrichment of cancer-related lncRNAs in such biological processes is reasonable. This finding validates the GO term in the rule.

Apart from Rules_66 and Rules_90 involving 70 GO terms, they also contribute to the identification of cancer-related lncRNAs. In addition to features that were already discussed, three parameters among the 70 GO terms were effective and essential for classification. GO: 2000052, which describes the activation (positive regulation) of the Wnt signaling pathway, could contribute to the identification of cancer-related lncRNAs. Considering the Wnt signaling pathway is essential in tumorigenesis. Therefore, the enrichment of cancer-related lncRNAs in this GO term is reasonable. Other terms, such as GO: 0010760 (describing the negative regulation of macrophage chemotaxis) (Snyderman and Pike, 1977; Roussos et al., 2011) and GO: 0033210 (describing the leptin-mediated signaling pathway) (Saxena et al., 2007; Wang et al., 2015) were also functionally correlated with cancer-associated biological processes and pathways.

### Rules for Cancer-Related lncRNA Exclusion

Among the 219 decision rules, 177 rules were designated for the exclusion of cancer-related lncRNAs. The majority of the rules contained too many parameters (GO terms or KEGG pathways) and were difficult to discuss. Without cancer specificity, such lncRNAs should be enriched in various general items, including essential biological processes for cells. Therefore, all such rules contribute to non-cancer-associated biological processes. To simplify, we selected two effective rules with few quantitative parameters to discuss. The detailed analyses can be seen below.

For Rule_2, the first parameter is also the general parameter GO: 0006275, which was already analyzed above. By contrast, this rule required high enrichment scores. The use of this GO term as a general marker for excluding cancer-related lncRNAs is reasonable. GO: 2000642 described the negative regulation of endosome transportation, which is a general biological process without the capacity of cancer recognition. Similar to the general GO terms, such as GO: 0006275 and GO: 0090162, Rules_3 also contributed to the exclusion of cancer-related lncRNAs. This rule used typical GO terms, such as GO:0050124 (*N*-acylneuraminate-9-phosphatase activity) and GO:0044795 (*trans*-Golgi network for the recycling of endosome transport). Both biological processes are non-cancer-specific processes without distinctive capacity for the identification of cancer-related lncRNAs. Therefore, the enrichment of lncRNAs’ co-expressed genes from non-cancer samples in such biological processes is reasonable.

## Conclusion

A wide and deep computational analysis was performed on cancer-related lncRNAs by presenting several decision rules. These rules indicated the combination of GO terms that could be a novel biomarker for determining cancer-related lncRNAs. We also tried our best to confirm the reliability of GO terms involved in the rules by review of literature. The new findings reported could bridge the novel connections between lncRNAs and cancers and provide novel insights about the diverse mechanisms of lncRNAs that participate in tumorigenesis.

## Data Availability Statement

The original contributions presented in the study are included in the article/Supplementary Material, further inquiries can be directed to the corresponding authors.

## Author Contributions

LZ contributed to the data analysis and interpretation, conception and design, and drafting of the manuscript. LY contributed to the data collection, conception, and design of the study. XY and RZ contributed to the data acquisition and analysis. All authors read and approved the final manuscript.

## Conflict of Interest

The authors declare that the research was conducted in the absence of any commercial or financial relationships that could be construed as a potential conflict of interest.

## Supplementary Material

Supplementary Material 1 mRMR feature list yielded through the mRMR method.

Supplementary Material 2 Performance of IFS incorporating DT when using different numbers of top features in the mRMR feature list.

Supplementary Material 3 Rules generated via DT with the top 970 features in the mRMR feature list.
